# The Dynamic Nature of Human Dermal Fibroblasts Is Defined by Marked Variation in the Gene Expression of Specific Cytoskeletal Markers

**DOI:** 10.3390/life12070935

**Published:** 2022-06-22

**Authors:** Akshay Kumar Ahuja, Luca Pontiggia, Ueli Moehrlen, Thomas Biedermann

**Affiliations:** 1 Tissue Biology Research Unit, Department of Surgery, University Children’s Hospital, University of Zurich, 8057 Zurich, Switzerland; luca.pontiggia@kispi.uzh.ch (L.P.); ueli.moehrlen@kispi.uzh.ch (U.M.); 2 Children’s Research Center, University Children’s Hospital Zurich, 8032 Zurich, Switzerland; 3 Faculty of Medicine, University of Zurich, 8057 Zurich, Switzerland

**Keywords:** human fibroblasts, gene expression, heterogeneity, single-cell transcriptomics, cytoskeletal markers

## Abstract

The evidence for fibroblast heterogeneity is continuously increasing, and recent work has shed some light on the existence of different sub-populations of fibroblasts in the human skin. Although we now have a more precise understanding of their distribution in the human body, we do not know whether their properties are predictive of where these cells derive from or whether these sub-types have functional consequences. In this study, we employed single-cell transcriptomics (10X Genomics) to study gene expression and segregate fibroblast sub-populations based on their genetic signature. We report the differential expression of a defined set of genes in fibroblasts from human skin, which may contribute to their dynamicity in vivo and in vitro. We show that the sub-population of fibroblasts expressing cytoskeletal markers, such as ANXA2, VIM, ACTB, are enriched in an adult skin sample. Interestingly, this sub-population of fibroblasts is not enriched in a neonatal skin sample but becomes predominant when neonatal fibroblasts are cultivated. On the other hand, the fibroblast sub-populations expressing COL1A1 and ELN are enriched in neonatal skin but are reduced in the adult skin and in fibroblasts from neonatal skin that are cultured in vitro. Our results indicate that fibroblasts are a dynamic cell type, and while their genetic make-up changes markedly, only a handful of genes belonging to the same functional pathway govern this alteration. The gene expression pattern of cytoskeletal markers may help in identifying whether the fibroblasts were isolated from an adult or an infant or whether they were cultivated, and this information could be useful for quality control in clinics and in cell banking. Furthermore, this study opens additional avenues to investigate the role of these markers in defining the complexity of human dermal fibroblasts.

## 1. Introduction

Fibroblasts are the principal cellular component of the connective tissue and provide a structural framework. They do so by producing extra-cellular matrix (ECM) components, including collagen, the most abundant protein in the human body. Fibroblasts are also involved in the regulation of biological processes, such as fibrosis, wound healing and cancer [1,2,3]. In particular, fibroblasts are an important constituent of skin and are found in its dermal compartment. Depending on their location in the dermis, fibroblasts exhibit different properties and functions [4,5,6,7]. Recent studies have shed light on the complexity and diversity of fibroblasts in human skin [8,9]; however, it remains unclear whether the body site location and/or age of the skin influence gene expression in this dynamic cellular population.

Single-cell transcriptomics (SCT) is a powerful tool to evaluate gene expression in a variety of cell types and species [10]. Methods have been refined over the past decade, and it is now possible to distinguish between two closely related cell types with a high accuracy [11]. SCT also allows for the evaluation of cellular heterogeneity within a given sample [12]. Indeed, SCT was employed to ascertain the marker expression and heterogeneous nature of human fibroblasts, respectively [8,9]. However, these studies presented certain shortcomings. Tabib et al. identified SFRP2 to be a major fibroblast marker based on data that effectively stemmed from a single cluster of cells obtained from one sample. Similarly, Philippeos et al. concluded that fibroblasts are heterogeneous after analysing <200 cells from the abdomen of a single sample. Hence, in this study we employed an unsupervised clustering approach and analysed three different samples to address the bias stemming from a single donor/single fibroblast population analysis.

We analysed a wide variety of markers that are associated with fibroblasts in this study. We compared the gene expression of fibroblasts derived from adult skin vs. neonatal skin vs. fibroblasts that were isolated from neonatal skin and cultivated. It is quite intriguing that we did not observe heterogeneity in the expression of cell surface markers (CD29 and CD164). However, we did find differences in the expression of extra cellular matrix factors (COL1A1 and ELN) and wound-healing markers (CXCL1 and TIMP1) among the different samples. What was unexpected is that cytoskeletal markers (ACTB, ANXA2 and VIM), which are not typically associated with fibroblasts, showed the strongest variation in expression across all the samples. These differences were noticed not only between the different samples, but also within each sample, which sheds light on the plasticity of fibroblasts. The main objective of our work was to study heterogeneity at the cellular level, which is why we analysed thousands of fibroblasts within each sample. Although further studies will be needed to validate our findings across multiple samples, our research opens new avenues to investigate cellular location and identity in more detail.

## 2. Results

**Figure 1:**
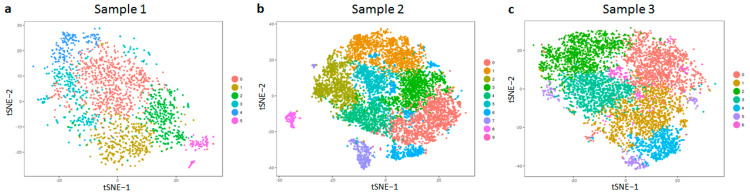
t-SNE clustering of fibroblasts on the basis of gene expression. Fibroblasts were isolated from the foreskin of a 1-year-old male donor (neonatal skin, (**a**)), thigh of a 52-year-old female donor (adult skin, (**b**)) and harvested after cultivating fibroblasts that were isolated from neonatal skin (**c**). All samples were subject to single-cell sequencing (see methods), and each sample was clustered into different sub-populations depending on the gene expression of individual cells.

The samples that we analysed in this study were chosen randomly to avoid selection bias: the first sample was the foreskin tissue of a 1-year-old boy (hereby called sample 1); the second sample was from the thigh of a 52-year-old woman (henceforth referred to as sample 2). Fibroblasts were isolated from both samples 1 and 2 and immediately processed for sequencing (see methods); a small fraction of fibroblasts from sample no. 1 was cultivated for six passages to study the effect of cell culture (named sample 3). Throughout this study, we analysed SCT data collected from these three different fibroblast samples. In order to decipher whether we could distinguish between subtypes of fibroblasts in these samples, we set out to perform unsupervised clustering without specifying which marker genes are commonly expressed by the cell type (Supplementary Figures S1–S3). We identified at least six distinct clusters of fibroblasts in each sample, which confirmed their heterogeneity (Figure 1a–c). Each cluster is defined by the expression levels of a particular subset of genes (Supplementary Figures S1–S3).

**Figure 2:**
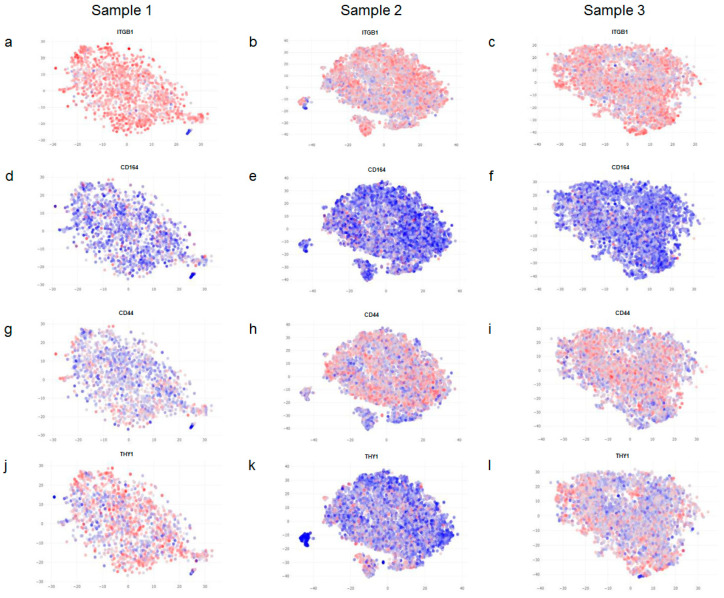
Comparison of known markers of fibroblasts. Fibroblasts from neonatal skin (**a**,**d**,**g**,**j**), adult skin (**b**,**e**,**h**,**k**) and neonatal fibroblasts cultivated for 6 passages (**c**,**f**,**i**,**l**) all exhibit uniform expression for ITGB1 or CD29 (**a**–**c**) and CD164 (**d**,**e**). A modest increase in the expression of CD44 is observed in adult fibroblasts and cultivated ones (**g**–**i**); whereas THY1 or CD90 expression is slightly lower in fibroblasts from adult skin (**k**) when compared to neonatal fibroblasts which are either freshly isolated (**j**) or cultivated (**l**). Blue denotes lower expression and red denotes higher expression of the indicated marker genes.

Next, we checked for the expression of known cell surface markers of fibroblasts. As can be observed in Figure 2, the expression levels of CD29 (ITGB1) and CD164 are quite uniform across all samples (Figure 2a–f). However, the expression of CD44 was observed to be marginally higher in samples 2 and 3 (Figure 2h,i) in comparison to sample 1 (Figure 2g), and CD90 (THY1) was found to be lower in sample 2 (Figure 2k) in comparison to levels observed in samples 1 and 3 (Figure 2j,l).

CD29 and CD164 served as important controls in our study and helped set up a baseline for the rest of our analysis since we could rule out any spurious effects arising due to differences in our samples with respect to variable parameters, such as body site location, age, sex, and cell culture. In other words, if body site location, age, sex, and cell culture influenced gene expression in general, we should have noticed a variation in CD29 and CD164 expression too, which was not the case.

**Figure 3:**
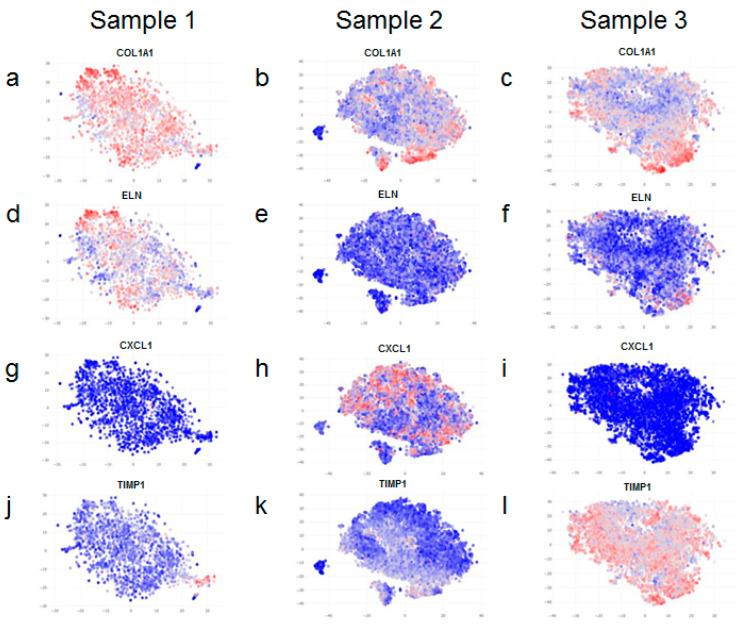
Differential gene expression of extra-cellular matrix factors and markers of wound healing. Fibroblasts from neonatal skin (**a**,**d**,**g**,**j**), adult skin (**b**,**e**,**h**,**k**) and neonatal fibroblasts cultivated for 6 passages (**c**,**f**,**i**,**l**) exhibit differential expressions for COL1A1 (**a**–**c**). Note that there is also a difference in COL1A1 expression between different clusters within each sample (more evident in (**b**,**c**)). ELN expression is reduced in fibroblasts from adult skin (**e**) and in cultivated neonatal fibroblasts (**f**) when compared to fibroblasts derived from neonatal skin (**d**). A substantial increase in the expression of CXCL1 is observed in fibroblasts derived from adult skin (**h**) when compared to neonatal fibroblasts that are freshly isolated (**g**) or cultivated (**i**), whereas there is little difference in TIMP1 expression in neonatal fibroblasts (**j**) and adult fibroblasts (**k**), and there is a significant increase in TIMP1 expression upon cultivation of neonatal fibroblasts (**l**). Blue denotes lower expression and red denotes higher expression of the indicated genes.

We then set out to see how the expression of extra-cellular matrix (ECM) factors was distributed in our samples. After careful analysis, we only observed a variation in the expression of the ECM factors COL1A1 and ELN. COL1A1 has a lower level of expression in samples 2 and 3 compared to sample 1 (Figure 3a–c); moreover, the expression of COL1A1 also varies between different clusters within individual samples (Figure 3b,c). ELN levels show the same trend as COL1A1, but the difference is more pronounced (Figure 3d–f). Next, we looked at the expression levels of wound-healing factors, CXCL1 and TIMP1. While CXCL1 levels are higher in sample 2, TIMP1 levels are higher in sample 3 compared to the other samples (Figure 3g–l).

**Figure 4:**
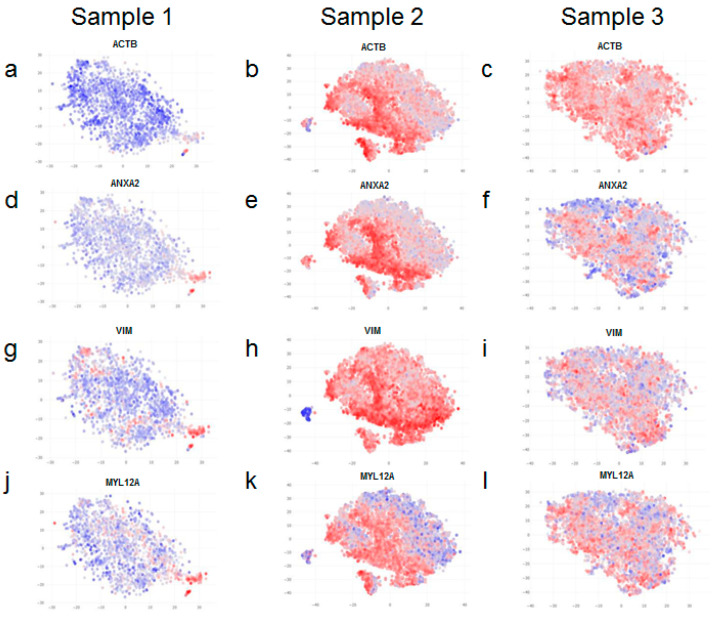
Over-expression of cytoskeletal and associated markers in adult fibroblasts and cultivated neo-natal fibroblasts. Fibroblasts from neonatal skin (**a**,**d**,**g**,**j**), adult skin (**b**,**e**,**h**,**k**) and neonatal fibroblasts cultivated for 6 passages (**c**,**f**,**i**,**l**) exhibit differential expression for ACTB (**a**–**c**), ANXA2 (**d**–**f**), VIM (**g**–**i**) and MYL12A (**j**–**l**). There is a substantial increase in the expression of all these genes in adult skin (**b**,**e**,**h**,**k**) and a modest increase upon cultivation of neonatal fibroblasts (**c**,**f**,**i**,**l**). Blue denotes lower expression and red denotes higher expression of the indicated genes.

Interestingly, we found most striking differences among the expression of cytoskeletal markers in the different samples. ACTB (Actin Beta) is involved in cell motility, intracellular signalling, and in the maintenance of the structure and integrity of the cell. Similarly, ANXA2 (Annexin A2) also plays a role in cell motility and is known to interact with the actin cytoskeleton. Vimentin (VIM) is a type III intermediate filament that comprises the cytoskeleton. Myosin light chain 12A (MYL12A) regulates cellular contraction and is also implicated in cell locomotion. Importantly, all of these genes show similar patterns of expression; their expression is markedly higher in samples 2 and 3 in comparison to sample 1 (Figure 4a–l).

**Figure 5:**
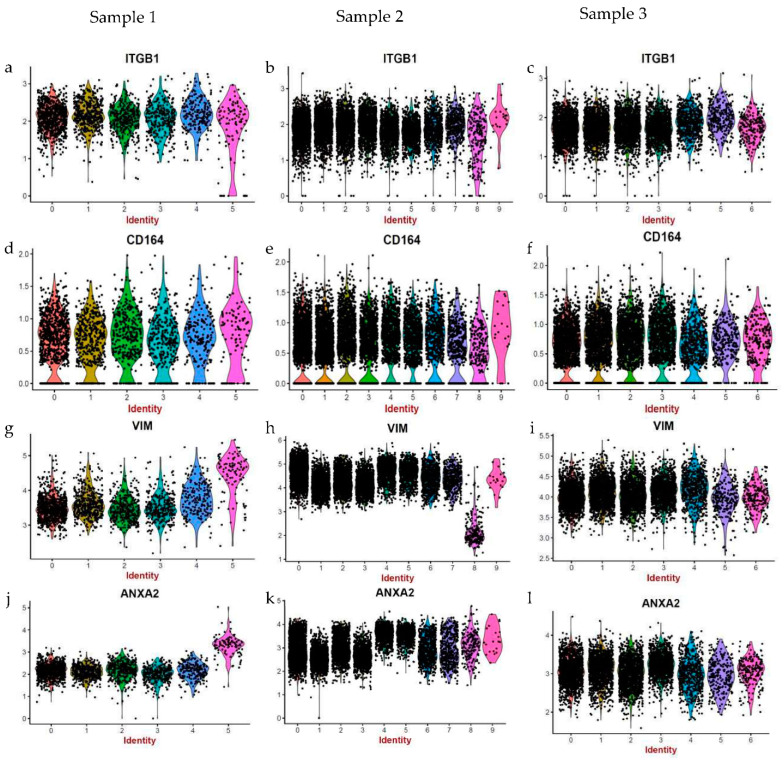
Heterogeneity in the expression of cytoskeletal and associated markers within samples. Fibroblasts from neonatal skin (**a**,**d**,**g**,**j**), adult skin (**b**,**e**,**h**,**k**) and neonatal fibroblasts cultivated for 6 passages (**c**,**f**,**i**,**l**) are homogenous in terms of expression of extra-cellular markers, such as ITGB1 or CD29 and CD164 ((**a**–**c**) and (**d**–**f**), respectively), i.e., the different sub-populations express similar levels of the aforementioned markers. However, each sample is heterogeneous for the expression of cytoskeletal and associated markers, such as Vimentin and ANXA2 ((**g**–**i**) and (**j**–**l**), respectively). Each colour represents a different sub-population clustered based on gene expression and each dot represents a cell.

A closer look revealed that only a certain proportion of fibroblasts within samples 2 and 3 (more evident in sample 2), but not all of them, express high levels of cytoskeletal markers (Figure 4b,e,h,k,c,f,i,l). Therefore, upon further analysis, we found clear evidence of intra-sample heterogeneity for a range of markers (Figure 5). Although fibroblasts within the same sample are homogenous with respect to expression of cell surface markers (Figure 5a–f), they do exhibit variation in the expression of cytoskeletal markers (Figure 5g–l).

## 3. Discussion

Fibroblasts produce ECM and are therefore an important part of any niche they occupy, especially since they provide the support and structure for other cell types in the body. Although the main function of this cellular population is well-defined, how each cell behaves also depends upon its location and interaction with neighbouring cells/tissue. Moreover, plasticity is a term that is synonymous with stem cells, but it is becoming clearer that differentiated cell types also exhibit dynamicity [12]. Fibroblast heterogeneity has remained an elusive concept and has recently been revisited by two independent groups [8,9]. However, reports of dermal fibroblasts showing variable behaviour have not been able to adequately address this unique aspect of cell biology.

It is natural to investigate factors that have, in the past, been associated with fibroblast receptor biology, remodelling, and wound healing [13,14,15,16]. However, upon careful analysis, we did not observe marked differences in the expression of cell surface markers in our samples. We did, instead, notice a downregulation of only COL1A1 and ELN in adult fibroblasts and after the prolonged cultivation of neonatal fibroblasts. Indeed, these data are consistent with a recently published study, which reports that fibroblasts produce lesser ECM as they age [17]. Additionally, among all of the wound healing factors, only CXCL1 and TIMP1 expression levels differed between the samples.

Our work also emphasizes the importance of using the right parameters and the appropriate controls to assess cellular heterogeneity. Many bioinformatic tools have been developed over the last decade, and this, combined with the access to surplus data, often makes it challenging to sieve through the noise in order to extract meaningful information. For instance, Tabib et al. report SFRP2 to be a major fibroblast marker, although SFRP2 was expressed in only one of the samples that they examined. In our study, we did not observe SFRP2 expression in freshly isolated and cultivated neonatal fibroblasts either; only a very tiny fraction of adult fibroblasts express SFRP2. Therefore, unlike CD29/ITGB1, which is expressed consistently across all our samples, SFRP2 does not appear to be a major fibroblast marker.

One of the key findings of our study is that fibroblasts are not heterogenous for all kinds of biomarkers. We did not notice any striking differences in cell surface marker expression between neonatal and adult fibroblasts. However, neonatal and adult fibroblasts show quite a distinct difference in expression levels of cytoskeletal markers. It remains to be established whether an increase in cytoskeletal marker expression with age has a functional consequence, but there have been reports that fibroblasts become more rigid as they age [18,19].

The increase in the expression of contractile markers may not necessarily translate into increased cellular contraction, but it is tempting to speculate that it may be the reason why human skin wrinkles with age. Although the differences in cytoskeletal gene expression between neonatal and adult fibroblasts may not be explicitly linked with aging due to other characteristic differences in the samples (such as body site location and sex), our results strongly suggest that the cultivation of fibroblasts exhibits the same trend (there is a marked increase in cytoskeletal gene expression when neonatal fibroblasts are cultivated), apparently mimicking physiological aging. Hence, our results suggest that cytoskeletal remodelling may be a function of physiological and induced aging.

Our work also sheds light on why there is a high degree of variability when reprogramming fibroblasts into induced pluripotent stem cells (iPSCs). If fibroblasts were to be homogenous, one would expect that each fibroblast would give rise to an iPSC clone with the same efficiency and regenerative potential. However, several studies report how reprogramming efficiency can vary across different experiments even when using the same batch of fibroblasts [20]. It would be interesting to explore whether fibroblasts expressing certain biomarkers would give rise to consistently stable iPSC colonies.

It is pertinent to mention that not only did we notice this difference in cytoskeletal gene expression between fibroblasts from different samples, but we also noticed striking differences in gene expression within fibroblasts derived from the same sample. Therefore, our data provide evidence of inter- and intra-sample fibroblast heterogeneity. The focus of this study was to investigate fibroblast heterogeneity at the cellular level; further studies will need to be carried out to expand this line of work and explore heterogeneity at the individual level.

In conclusion, our research demonstrates that SCT is a powerful tool that can potentially be used to distinguish fibroblast samples from one another for parameters such as age, body location, and sex, and to identify different fibroblast sub-populations within a given sample. These data can have important implications in cell banking and quality control in the field of regenerative medicine, and future studies may be designed to assess if our findings can be observed across multiple samples.

## 4. Materials and Methods

### 4.1. Fibroblast Isolation and Cultivation

The investigation was conducted according to the Declaration of Helsinki principles and after acceptance by the Ethic Commission of the Canton Zurich. Children’s parents or patients gave informed consent to the use of their skin samples.

Skin samples (foreskin from a 1-year-old boy and thigh biopsy from a 52-year-old woman, approximately 2.5 cm^2^ each) were digested overnight with dispase (purchased from Thermo Fisher Scientific, Basel, Switzerland). The epidermis was separated from the dermis mechanically (using forceps) and discarded. The dermis was then cut into small pieces and digested with collagenase for 1 h. The digested dermis was passed through a cell strainer to get rid of clumps and the dermal fraction was plated onto a tissue culture dish containing a fibroblast medium (DMEM and 10% FBS with 0.1% Gentamycin; purchased from Thermo Fisher Scientific). The medium was changed every 2 days. Fibroblasts were trypsinized 6 days after seeding, neutralized, and counted (on the day of the RNA sequencing experiment). Some fibroblasts from the foreskin of the 1-year-old donor were retained and cultured for 6 passages (approx. 5 weeks).

### 4.2. Single-Cell RNA Sequencing Using 10X Genomics Platform

The quality and concentrations of the single-cell preparations were evaluated using a haemocytometer and adjusted to 1000 cells/µL. Then, 10,000 cells per sample were loaded into a 10X Chromium controller, and library preparation was performed according to the manufacturer’s indications (single-cell 3′ V2 protocol, 10X Genomics, Pleasanton, CA, USA). The resulting libraries were sequenced in an Illumina NextSeq500 sequencer according to 10X Genomics recommendation to a depth of around 50,000 reads per cell.

### 4.3. Single-Cell RNA Sequencing Data Analysis

De-multiplexing, collapsing of unique molecular identifiers (UMIs), and alignment of reads to the human transcriptome (GRCh38 Ensembl release 91) were performed using the Cellranger toolkit (version 2.0.2, 10X Genomics) provided by 10X Genomics. For the three samples (foreskin fibroblasts of a 1-year-old male donor, cultivated fibroblasts isolated from the 1-year-old donor for 6 passages, and fibroblasts from the thigh of a 52-year-old female donor), 2207, 6571, and 9251 cells, respectively, were profiled.

A further downstream analysis was performed with Seurat R package (version 4.0, New York, NY, USA) (http://satijalab.org/seurat/, accessed on 1 February 2021). To exclude low-quality cells and duplets, cells for which fewer than 1500 genes or higher than 7500 genes were detected were filtered out. All genes that were not detected in at least 10 cells were also discarded in the respective analysis. Library-size normalization was carried out for the UMI-collapsed gene expression values for each cell barcode by scaling the total number of transcripts and multiplying by 10,000. The data were then natural-log transformed for all downstream analyses. The detection of highly variable genes was carried out based on the average expression and dispersion for each gene such that genes were placed into bins, and then z-scores for dispersion were calculated within each bin, as described in [12].

The dimensionality of the dataset was reduced using principal component analysis (PCA). To identify statistically significant principal components (PCs), a modified randomization approach (‘jack straw’), as described in [12], was used at first. Then, 20 PCs for downstream analyses were selected for each sample. Unbiased clustering of the cells was performed using a shared nearest neighbour (SNN) modularity optimization-based clustering algorithm, as implemented in the FindClusters function in Seurat. Setting a resolution of 0.4 identified 4 cell populations. Differentially expressed genes (markers) for each of the cell populations were determined using Wilcoxon rank sum test.

## Data Availability

Data available on request.

## Supplementary Materials

The following supporting information can be downloaded at: https://www.mdpi.com/article/10.3390/life12070935/s1, Figure S1: Sequencing summary of sample no. 1 (1-year-old donor). Figure S2: Sequencing summary of sample no. 2 (52-year-old donor). Figure S3: Sequencing summary of sample no. 3 (fibroblasts isolated from 1-year-old donor and cultivated for 6 passages).
