# Supplementary material for: The Dynamic Nature of Human Dermal Fibroblasts Is Defined by Marked Variation in the Gene Expression of Specific Cytoskeletal Markers
Source: Life (Basel). 2022 Jun 22;12(7):935. doi: 10.3390/life12070935 (PMC9319478; doi:10.3390/life12070935)
Supplement: Supplementary file 1 [file life-12-00935-s001.zip › life-1787841-supplementary.pdf]

Estimated Number of Cells

2,207

Mean Reads per Cell	Median Genes per Cell
62,671	4,866

Sequencing

Number of Reads	138,314,946
Valid Barcodes	98.1%
Reads Mapped Confidently to Transcriptome	65.7%
Reads Mapped Confidently to Exonic Regions	70.0%
Reads Mapped Confidently to Intronic Regions	21.3%
Reads Mapped Confidently to Intergenic Regions	1.8%
Reads Mapped Antisense to Gene	5.3%
Sequencing Saturation	40.9%
Q30 Bases in Barcode	97.6%
Q30 Bases in RNA Read	85.6%
Q30 Bases in Sample Index	97.4%
Q30 Bases in UMI	97.8%

Cells

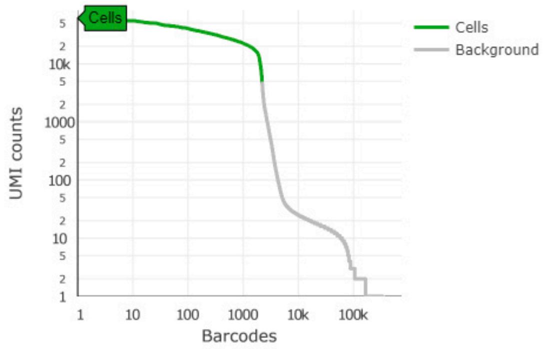

Estimated Number of Cells	2,207
Fraction Reads in Cells	94.8%
Mean Reads per Cell	62,671
Median Genes per Cell	4,866
Total Genes Detected	23,408
Median UMI Counts per Cell	21,476

Sample

Name	p2375_1yo
Description	
Transcriptome	10X_Ref_Human_GRCh38.p10_20180305_Release_91
Chemistry	Single Cell 3' v2
Cell Ranger Version	2.0.2

Supplementary Figure S1: Sequencing summary of sample no. 1 (1-year-old donor)

Estimated Number of Cells

9,251

Mean Reads per Cell	Median Genes per Cell
39,720	3,644

Sequencing

Number of Reads	367,458,077
Valid Barcodes	96.4%
Reads Mapped Confidently to Transcriptome	65.7%
Reads Mapped Confidently to Exonic Regions	69.1%
Reads Mapped Confidently to Intronic Regions	11.8%
Reads Mapped Confidently to Intergenic Regions	1.5%
Reads Mapped Antisense to Gene	5.8%
Sequencing Saturation	25.6%
Q30 Bases in Barcode	88.8%
Q30 Bases in RNA Read	67.3%
Q30 Bases in Sample Index	84.7%
Q30 Bases in UMI	87.1%

Cells

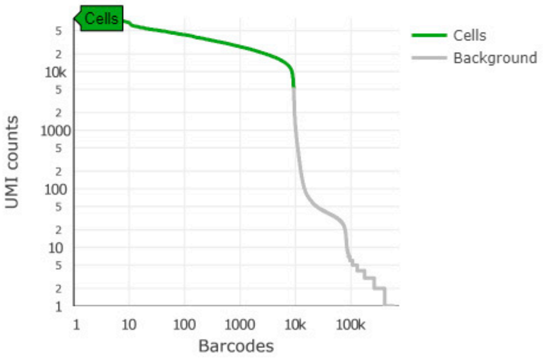

Estimated Number of Cells	9,251
Fraction Reads in Cells	96.2%
Mean Reads per Cell	39,720
Median Genes per Cell	3,644
Total Genes Detected	25,760
Median UMI Counts per Cell	16,922

Sample

Name	P0_Fibroblasts_52yo_thigh
Description	
Transcriptome	10X_Ref_Human_GRCh38.p10_20180305_Release_91
Chemistry	Single Cell 3' v2
Cell Ranger Version	2.0.2

Supplementary Figure S2: Sequencing summary of sample no. 2 (52-year-old donor)

Estimated Number of Cells

6,571

Mean Reads per Cell	Median Genes per Cell
**52,429**	**4,346**

Sequencing

Number of Reads	344,514,553
Valid Barcodes	96.5%
Reads Mapped Confidently to Transcriptome	66.7%
Reads Mapped Confidently to Exonic Regions	70.0%
Reads Mapped Confidently to Intronic Regions	10.3%
Reads Mapped Confidently to Intergenic Regions	1.4%
Reads Mapped Antisense to Gene	5.7%
Sequencing Saturation	28.9%
Q30 Bases in Barcode	89.0%
Q30 Bases in RNA Read	67.2%
Q30 Bases in Sample Index	87.7%
Q30 Bases in UMI	87.2%

Cells

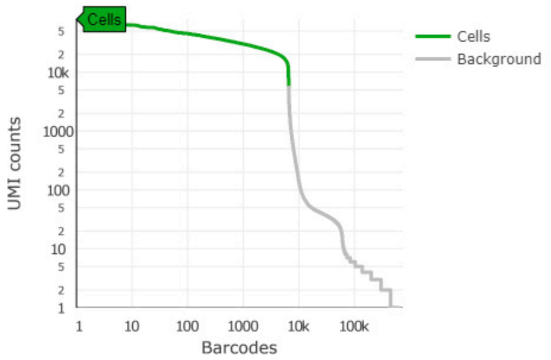

Estimated Number of Cells	6,571
Fraction Reads in Cells	96.5%
Mean Reads per Cell	52,429
Median Genes per Cell	4,346
Total Genes Detected	24,175
Median UMI Counts per Cell	22,239

Sample

Name	P6_Fibroblasts_Foreskin
Description	
Transcriptome	10X_Ref_Human_GRCh38.p10_20180305_Release_91
Chemistry	Single Cell 3' v2
Cell Ranger Version	2.0.2

Supplementary Figure S3: Sequencing summary of sample no. 3 (fibroblasts isolated from 1-year-old donor and cultivated for 6 passages)
